# Supplementary material for: Age-related differences in psychopathology within sex chromosome trisomies
Source: Eur Child Adolesc Psychiatry. 2025 May 23;34(10):3275–84. doi: 10.1007/s00787-025-02743-4 (PMC12592274; doi:10.1007/s00787-025-02743-4)
Supplement: Supplementary file 1 — Supplementary file1 (DOCX 189 KB) [file 787_2025_2743_MOESM1_ESM.docx]

**SUPPLEMENTARY TABLES AND FIGURES**

**Title:** Age-related differences in psychopathology within sex chromosome trisomies

**Authors:** Melissa R. Roybal (melissa.roybal@nih.gov)^1^, Siyuan Liu (siyuan.liu@nih.gov)^1^, Isabella G. Larsen (bella.larsen@nih.gov, OrcID: 0000-0001-8520-5539)^1^, Anastasia Wass (a.c.e.wass@gmail.com)^2^, Lukas Schaffer (luke.schaffer@gmail.com)^3,4^, Tiffany Ajumobi (tajumobi@gmail.com)^5^, Ethan T. Whitman (ethan.whitman@gmail.com)^6^, Allysa Warling (allysawarling@gmail.com, OrcID: 0000-0001-9412-6357)^7^, Liv Clasen (clasenl@mail.nih.gov)^1^, Jonathan Blumenthal (jonathanblumenthal@mail.nih.gov)^1^, Srishti Rau (srrau@childrensnational.org)^8^*, Armin Raznahan (raznahana@mail.nih.gov)^1^*

^1^ Section on Developmental Neurogenomics, National Institute of Mental Health, National Institutes of Health, Bethesda, MD, United States

^2^ Georgetown University School of Medicine, Washington, D.C., United States

^3^ Institute for Behavioral Genetics, University of Colorado Boulder, Boulder, CO, United States

^4^ Department of Psychology and Neuroscience, University of Colorado Boulder, Boulder, CO, United States

^5^ School of Medicine, The Johns Hopkins University, Baltimore, MD, United States

^6^ Department of Psychology & Neuroscience, Duke University, Durham, NC, United States

^7^ Harvard Medical School, Boston, MA, United States

^8^ Children’s National Health System, Center for Autism Spectrum Disorders and Division of Neuropsychology, Washington, D.C., United States

*These authors contributed equally to this work.

**Corresponding author identification and email address:**Armin Raznahan [raznahana@mail.nih.gov](mailto:raznahana@mail.nih.gov)

|  |  |  |  |  |  |  | **Group Comparisons** | | |
| --- | --- | --- | --- | --- | --- | --- | --- | --- | --- |
|  | **Total** | **XX** | **XY** | **XXX** | **XXY** | **XYY** | **Comparing all groups** | **SCTs vs. controls** | **Comparing SCT groups only** |
|  |  |  |  |  |  |  |  |  |  |
| **Federal Race Category: N(%)** |  |  |  |  |  |  |  |  |  |
| Asian | 11 (2.2) | 3 (2.0) | 3 (2.0) | 3 (6.0) | 1 (1.1) | 1 (1.7) | *χ*2= 35.87* | *χ*2= 34.99** | *χ*2= 9.94 |
| Black | 34 (6.7) | 17 (11.3) | 13 (8.5) | 0 (0) | 4 (4.3) | 0 (0) |  |  |  |
| American Indian / Alaska Native | 1 (0.2) | 0 (0) | 0 (0) | 0 (0) | 1 (1.1) | 0 (0) |  |  |  |
| White | 424 (84) | 123 (81.5) | 120 (78.4) | 44 (88.0) | 81 (88.0) | 56 (95.0) |  |  |  |
| More than one race | 33 (6.5) | 6 (4.0) | 17 (11.1) | 3 (6.0) | 5 (5.4) | 2 (3.4) |  |  |  |
| Unknown / not reported | 2 (0.4) | 2 (1.3) | 0 (0) | 0 (0) | 0 (0) | 0 (0) |  |  |  |
|  |  |  |  |  |  |  |  |  |  |
| **Federal Ethnicity Category: N(%)** |  |  |  |  |  |  |  |  |  |
| Hispanic | 34 (6.7) | 10 (6.6) | 6 (4) | 4 (8) | 9 (9.8) | 5 (8.5) | *χ*2= 9.23 | *χ*2= 3.60 | *χ*2= 2.58 |
| Non-Hispanic | 468 (92.7) | 141 (93.4) | 146 (95.4) | 46 (92) | 81 (88) | 54 (91.5) |  |  |  |
| Unknown | 3 (0.6) | 0 (0) | 1 (0.7) | 0 (0) | 2 (2.2) |  |  |  |  |

**Supplementary Table 1 Additional demographic variables** * *p* < .05, ***p* < .001

| **CBCL Scale** | **CBCL T-score by group, M(SD)** | | | | |
| --- | --- | --- | --- | --- | --- |
|  | **XX** | **XY** | **XXX** | **XXY** | **XYY** |
|  |  |  |  |  |  |
| anxdep | 51 (0.5) | 51.3 (0.5) | 60.3 (1.1)* | 57.9 (0.8)* | 59.7 (1.0)* |
| withdep | 51.5 (0.5) | 52.0 (0.5) | 61.7 (1.1)* | 58.9 (0.8)* | 61.3 (1.0)* |
| somatic | 52.2 (0.5) | 52.3 (0.5) | 62.6 (1.1)* | 59.2 (0.9)* | 63.3 (1.0)* |
| social | 50.6 (0.5) | 51.2 (0.5) | 63.5 (1.0)* | 60.7 (0.8)* | 68.1 (0.9)* |
| thought | 51.1 (0.5) | 51.7 (0.5) | 60.5 (1.0)* | 60.0 (0.8)* | 66.0 (1.0)* |
| attention | 50.9(0.5) | 51.4 (0.5) | 67.1 (1.1)* | 60.9 (0.8)* | 68.6 (1.0)* |
| rulebreak | 51.3 (0.4) | 51.4 (0.4) | 56.6 (0.9)* | 56.1 (0.7)* | 59.0 (0.8)* |
| aggressive | 50.7 (0.5) | 51.0 (0.5) | 58.1 (1.0)* | 56.5 (0.8)* | 62.6 (0.9)* |
| internal | 42.5 (0.8) | 43.6 (0.8) | 61.7 (1.6)* | 57.6 (1.3)* | 62.2 (1.5)* |
| external | 42.0 (0.8) | 42.6 (0.8) | 53.9 (1.6)* | 53.8 (1.2)* | 59.4 (1.5)* |
| totalprob | 40.0 (0.8) | 41.3 (0.7) | 61.4 (1.6)* | 58.7 (1.2)* | 65.8 (1.4)* |

**Supplementary Table 2 Mean CBCL T-score for each CBCL dimension in each karyotype group (estimated at mean age of the combined cohort).** Asterisk denotes Tukey post hoc adjusted *p*-value for comparison of each SCT group’s CBCL T-score distribution relative to their respective gonadal control group (*p* < .05).

**
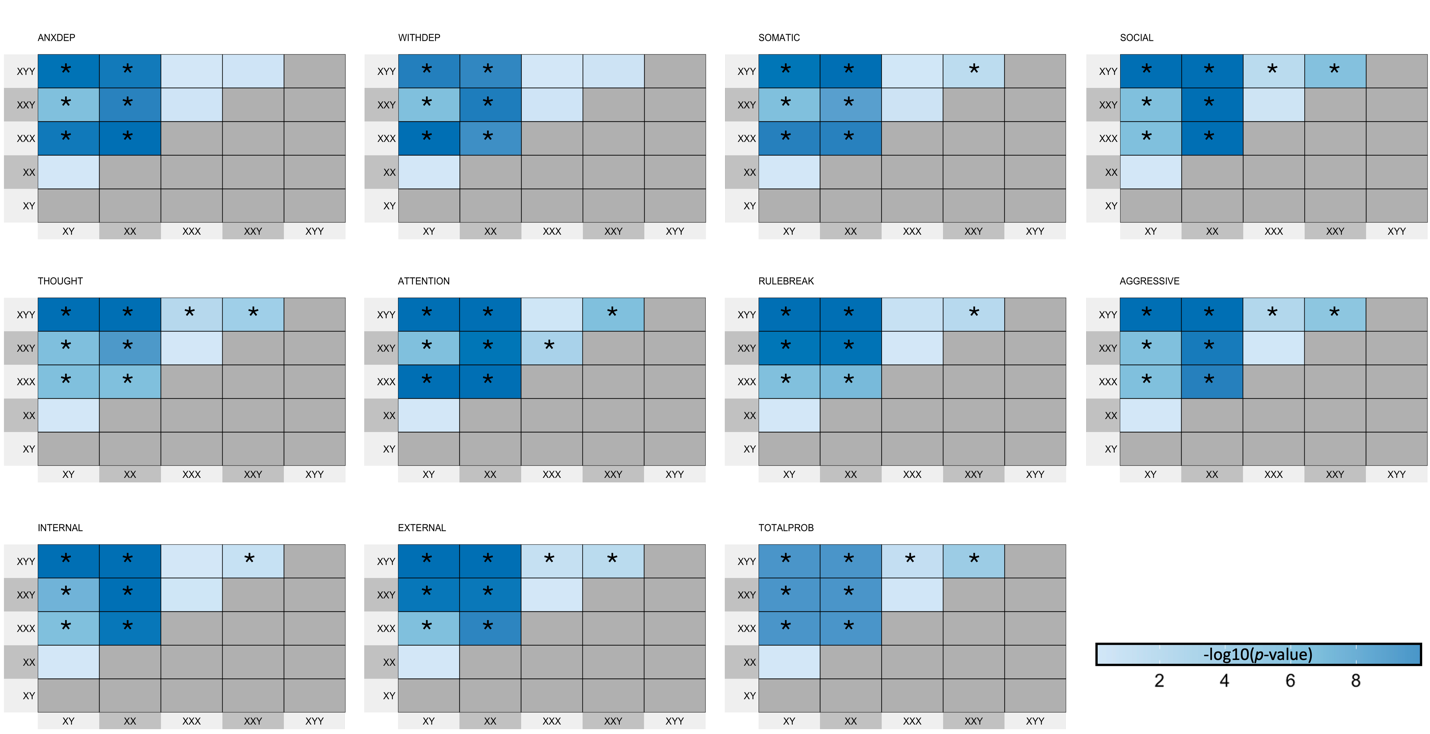
Supplementary Fig. 1 Pairwise tests for inter-group differences in mean CBCL T-score for each CBCL dimension.** Colors encode the -log10(*p*-value) from Tukey test for each group difference. Asterisks denote statistically significant contrasts (adjusted *p*-value < .05). Directions of group differences are detailed in Supplementary Table 2.
